# Importance of *NUDT15* Polymorphisms in Thiopurine Treatments

**DOI:** 10.3390/jpm11080778

**Published:** 2021-08-10

**Authors:** Yoichi Tanaka, Yoshiro Saito

**Affiliations:** Division of Medicinal Safety Science, National Institute of Health Sciences, Kawasaki City 210-9501, Kanagawa, Japan; yoshiro@nihs.go.jp

**Keywords:** *NUDT15*, thiopurine, mercaptopurine, azathioprine, Asian, adverse effect, implementation

## Abstract

Thiopurines, mercaptopurine, and azathioprine are used as immunosuppressants in the treatments of inflammatory bowel disease, rheumatoid arthritis, and organ transplantation and as chemotherapeutic drugs for the treatment of acute leukemia and chronic myeloid leukemia. This drug class sometimes causes severe adverse reactions, including bone marrow suppression and hair loss. Genetic polymorphisms of the metabolizing enzyme thiopurine S-methyltransferase have been used for predicting these reactions in Caucasians, but these allele frequencies are less frequently observed in Asian populations. Recently, nudix hydrolase 15 (*NUDT15*) polymorphisms have been shown to play an important role in thiopurine-induced adverse reactions in Asians. In this review, we summarize the *NUDT15* studies, mainly in Asian countries, and their implementation in several countries.

## 1. Introduction

Thiopurines, 6-mercaptopurine (6-MP) and azathioprine (AZA) are used as immunosuppressive and cytotoxic agents. AZA is used to maintain remission of inflammatory bowel disease (IBD) [1,2] and rheumatoid arthritis [3] and to inhibit immunological rejection of transplanted organs [4]. In hematological malignancies, 6-MP is used as maintenance therapy for 2–3 years [5]. Although thiopurine is the main component of these therapies, severe adverse effects of grade 3 or higher frequently occur. The typical adverse effects of thiopurines include myelosuppression, hair loss, hepatotoxicity, and pancreatitis.

**Figure 1:**
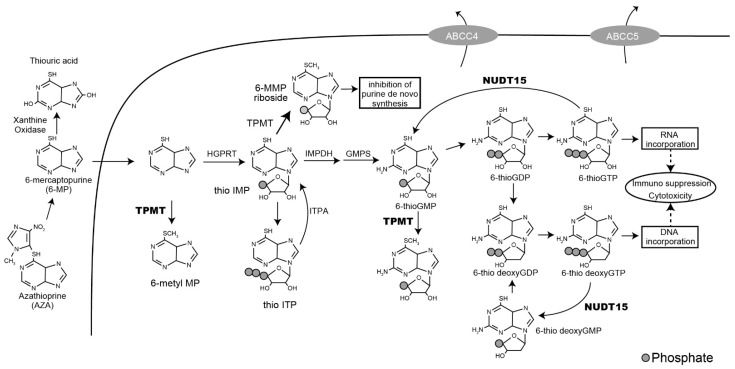
Thiopurine metabolism pathway. ABCC, ATP-binding cassette, subfamily C; GDP, guanosine diphosphate; GMP, guanosine monophosphate; GMPS, guanine monophosphate synthetase; GTP, guanosine triphosphate; HGPRT, hypoxanthine guanine phosphoribosyltransferase; IMP, inosine monophosphate; ITP, inosine triphosphate; IMPDH, inosine monophosphate dehydrogenase; *NUDT15*, nudix hydrolase 15; TPMT, thiopurine S-methyltransferase.

To date, many studies have used a genetic approach to predict thiopurine-induced severe toxicities, especially myelosuppression. Thiopurine S-methyltransferase (TPMT) is an enzyme involved in thiopurine metabolism (Figure 1), and its genetic polymorphisms are known to be risk factors for intolerance to thiopurines [6]. The TPMT variants of c.238G>C, c.460G>A, and c.719G>A induce protein instability and decrease the TPMT enzyme activity [7]. A low TPMT activity results in the accumulation of active metabolites, thiopurine nucleosides, in the cells, leading to cytotoxicity. The frequencies of variants with low TPMT activity differ among ethnic groups [6]. Caucasians and Africans have a high frequency of these polymorphisms, but Asians have lower frequencies of these *TPMT* nonfunctional variants; nonetheless, the frequencies of severe toxicities are similar among these races.

In addition to TPMT polymorphisms, nudix hydrolase 15 (*NUDT15*) c.415C>T (p.R139C) was associated with thiopurine intolerance in Asian populations, as reported in a previous genome-wide association study (GWAS) [8,9]. To date, *NUDT15* is known to have 26 alleles, and *NUDT15* *2 (p.V18_V19insGV and c.415C>T), *3 (c.415C>T), and *9 (c.50delGAGTCG) are recognized as loss-of-function variants (Table 1) [10,11]; however, other variants have been uncertain about the functional significance, referring to the Pharmacogene Variation Consortium (PharmVar, www.pharmvar.org). The *NUDT15* enzyme dephosphorylates thiopurine triphosphate, which is the active metabolite incorporated into the DNA, to its monophosphate (Figure 1). The *NUDT15* loss-of-function variants induce the increased levels of thiopurine triphosphate and thus higher incorporation of thioguanine nucleotides into the DNA and RNA. As a result, patients who inherited these variants experienced severe thiopurine-induced cytotoxicities, such as myelosuppression and alopecia. *NUDT15* c.415C>T (p.R139C) is commonly detected in Asians but is rarely found in Caucasians and Africans, with allele frequencies of 0.12 and <0.01, respectively [6]. In this review, we aimed to provide a summary of the associations of *NUDT15* alleles and their clinical significance, mainly in Asians, as well as their implementation in several countries.

## 2. *NUDT15* Variants and Toxicities in Thiopurine Metabolism

### 2.1. Thiopurine for Inflammatory Bowel Disease

Thiopurine is administered for maintenance of remission and is used as a steroid-sparing treatment for IBD. Approximately 25% of patients experience thiopurine-induced toxicities, especially myelosuppression [18]. The first study that reported the association between *NUDT15* polymorphisms and 6-MP-induced toxicities was the GWAS conducted in Korean patients with Crohn’s disease. Genotyping was performed in 978 patients using the immunochip array, while genotype imputation was carried out using the Asian reference panel. This study identified that the *NUDT15* c.415C>T (p.R139C) variant was strongly associated with thiopurine-induced early leukopenia (white blood cell [WBC] count <3000/mm^3^) (odds ratio [OR] = 35.6; P [combined] = 4.88 × 10^−94^) [8]. The association between the c.415C>T variant and with early leukopenia was replicated, and severe hair loss occurred within 8 weeks in 142 Japanese IBD patients with homozygous T variants treated with thiopurine [19]. The *NUDT15* enzyme with 139C showed deficient activity and was significantly associated with thiopurine-induced leukopenia and alopecia. Some Asian study groups have validated the association between p.R139C or other variants of *NUDT15* and thiopurine-induced toxicities in patients with IBD. The effect of multiple *NUDT15* variants, c.415C>T, c.55_56insGAGTCG, and c.52G>V, were evaluated to determine its association with leukopenia in 732 Chinese patients with IBD [12]. Results showed that these three variants were significantly associated with all grades of leukopenia, and a combination of these variants increased predictivity for leukopenia. By contrast, another study reported that *NUDT15* functional variants in exon 1, c.55_56insGAGTCG a and c.52G>V, were not significantly associated with leukopenia and hair loss in Japanese patients with IBD [20].

The optimal therapeutic strategy using pharmacogenetic factors for the prediction of thiopurine-induced leukopenia and alopecia was retrospectively evaluated in 970 Japanese patients with IBD [13]. This study divided the *NUDT15* diplotypes into different categories to estimate the enzyme activity (normal-normal, normal-intermediate, normal-low, intermediate-low, and low-low) and showed that the categorized diplotypes were significantly associated with leukopenia but not with severe alopecia. To determine the best method to predict severe leukopenia and alopecia, they performed GWAS and showed that *NUDT15* c.415C>T had the strongest association with severe leukopenia and alopecia (*p* = 1.3 × 10^−33^ and 4.3 × 10^−29^, respectively), while previouslyreported variants of *TPMT*, *ABCC4*, and *RUNX1* were not associated with. *NUDT15* c.416G>A (p.R139H, *4) was a rare variant, and Cys/His at codon 139 was a risk factor for acute severe leukopenia (within 8 weeks after thiopurine treatment) but not for alopecia. In a previous case report, patients with c.416AA experienced thiopurine-associated severe leukopenia and alopecia [17].

Thiopurine dose must be reduced in patients with *NUDT15* variants to avoid thiopurine-induced severe toxicities, and therapeutic efficacy must be obtained at low doses. Some previous studies have shown an association between thiopurine dose and its efficacy in IBD patients with *NUDT15* variants. Maeda et al. reported thiopurine-induced toxicities and efficacy in 30 patients with *NUDT15* c.415 C/T [21]. These heterozygous patients were treated with 0.25 mg/kg/day of 6-MP, which was lower than the dose administered in c.415C/C patients (0.48 mg/kg). The non-relapse rates in ulcerative colitis treated with thiopurine monotherapy and surgery-free rates in Crohn’s disease treated with combination therapy (thiopurines and antitumor necrosis factor-α agents) for maintenance of remission were not significantly different between c.415C/C patients and C/T patients at 60 months (*p*= 0.339 and *p* = 0.422, respectively). Xu et al. showed that patients with heterozygous p.R139C were administered with significantly lower AZA dose compared with those with wild-type variants (0.83 mg/kg vs. 1.04 mg/kg), but the rate of clinical remission did not differ between these two patient groups [22]. Further studies are needed to determine the recommended dose for IBD patients with the *NUDT15* variant; however, these findings might support the use of reduced thiopurine dose as initial treatment for patients with *NUDT15* variants.

### 2.2. 6-Mercaptopurine for Acute Lymphoblastic Leukemia

6-MP is one of the primary components of multidrug therapy for acute lymphoblastic leukemia (ALL) [5]. In maintenance therapy, 6-MP is administered orally along with weekly methotrexate therapy for 2–3 years; a lower 6-MP dose intensity is associated with worse therapeutic outcomes [23].

In childhood ALL, the *NUDT15* allele was initially associated with the administration of a 6-MP tolerance dose within the first 6 months in a multiracial GWAS of 657 children with ALL in the AALL03N1 study [9]. *TPMT* rs1142345 and *NUDT15* c.415C>T (rs116855232) were significantly associated with the 6-MP dose in the initial 6 months of maintenance therapy. In this study, the *NUDT15* variant allele was common among Asians and Hispanics, and this variant affected the 6-MP dose intensity with effective power similar to that of the *TPMT* variant. We evaluated the association between *NUDT15* variant p.R139C and 6-MP intolerance in 92 Japanese children with ALL who received maintenance therapy [16]. Although the standard dose of 6-MP for Asians is 40–50 mg/m^2^, patients with *NUDT15* R139C frequently experienced leukopenia (WBC count <2000/mm^3^), and all of those with c.415T/T (p.139C/C) genotype developed leukopenia within 60 days. In this study, the 6-MP average doses of 40.7, 29.3, and 8.8 mg/m^2^ were used as maintenance therapy for patients with the c.415C/C, C/T, and T/T genotypes, respectively. These associations were validated in Thai [24,25], Taiwanese [26] and Korean populations [27]. In Chinese 60 ALL children whose 6-MP initial doses were adjusted based on *TPMT* genotypes, *NUDT15* c.415C>T variant was also one of the significant risk factors for thiopurine-induced leukopenia (WBC ≤ 2000/mm^3^) [28]. Thus, *NUDT15* c.415T/T is one of the major risk factors for severe leukopenia during early maintenance therapy, and 6-MP treatment is associated with a dramatic decrease in neutrophil counts. In 100 Thai children with ALL, those with mono- or bi-allelic variants of *NUDT15* c.415C>T and c.55_56insGAGTCG showed lower neutrophil counts compared with those with wild-type variants, although the adjusted doses during the first 6 months of maintenance therapy for *NUDT15* wild-type, mono-allelic, and bi-allelic variants were 50.0, 36.6, and 12.5 mg/m^2^/day, respectively [29]. In 6-MP treatment, patients with bi-allelic variants experienced severe bone marrow suppression and required the discontinuation or reduction of significant doses. We recently evaluated the association between 6-MP intolerability and clinical characteristics in 37 children with ALL bearing *NUDT15* bi-allelic variants in an Asian international collaboration retrospective study [14]. Among those with *NUDT15* bi-allelic variants, more than 90% of the patients had a WBC count of <2000/mm^3^ or a neutrophil count of <1000/mm^3^. Interestingly, 86% of the patients had a neutrophil count of <500/mm^3^. Our data showed that careful monitoring of neutrophil counts was of utmost importance, and the tolerable 6-MP dose was only 5.2 mg/m^2^ to maintain sufficient leukocyte range during maintenance therapy for patients with *NUDT15* variants. We further demonstrated that adjusting the initial 6-MP dose is important to prevent the discontinuation of treatment and to allow the continuation of maintenance therapy, based on the *NUDT15* genotype. In patients with *NUDT15* bi-allelic variant, those who received an initial 6-MP dose of less than 10 mg/m^2^ experienced a shorter duration of therapy interruption than those who received more than 10 mg/m^2^ in the first 8 weeks (median range, 0 vs. 16 days) [14]. Based on the guideline of the Clinical Pharmacogenetics Implementation Consortium (CPIC), an initial 6-MP dose of 10 mg/m^2^ is recommended for patients with *NUDT15* poor metabolizer diplotypes (*2/*2, *2/*3, and *3/*3) (Table 2) [6]. Thus, our findings mentioned above verified this recommendation. To prevent the dramatic decline in neutrophil count, reducing the initial 6-MP dose to less than 10 mg/m^2^ is important, although a 6-MP dose adjustment is still recommended based on the patients’ leucocyte count during the treatment.

*NUDT15* alleles bearing variations other than those at codon 139 have uncertain functional significance for 6-MP tolerability in ALL patients because these variants are considered rare. In our study, eight patients inherited non-codon 139 variant alleles. The average 6-MP dose in patients with *2/*5, *2/*7, and *3/*5 diplotypes was less than 10 mg/m^2^, while that in patients with *2/*6 and *5/*5 were tolerable for more than 10 mg/m^2^ [14]. A patient with *5/*5 required a reduction in the 6-MP dose because of the decreasing WBC counts, but a tolerable 6-MP dose of 17 mg/m^2^ was administered to maintain suitable blood cell counts. In another study conducted in Taiwanese children with ALL, the 6-MP dose for patients with *3/*6 and *2/*7 genotypes was less than 10 mg/m^2^ [15]. The frequencies of variants in exon 1 (such as c. 55_56insGAGTCG and c.52G>V) are extremely low, and the effects of these variants have not been revealed. To the best of our knowledge, the tolerable dose of 6-MP differs in various *NUDT15* alleles.

### 2.3. Other Diseases

AZA has been used for the treatment of autoimmune and rheumatic diseases; however, only a few observational studies and case reports have examined patients with these diseases who developed AZA-induced toxicities associated with the *NUDT15* variants.

In 149 Chinese patients with autoimmune hepatitis, the *NUDT15* c.415T allele was significantly associated with leukopenia and neutropenia [30]. The maintenance dose of AZA for patients with the *NUDT15* c.415T variant was lower than that of patients with the wild-type variant. The maintenance AZA doses for patients with the C/C, C/T, and T/T diplotypes were 1.23, 0.96, and 0.2 mg/kg/day, respectively. Regarding rheumatic disease, NUDT15*3 genotype was associated with AZA-induced hematological toxicities, gastrointestinal effects, and hypochromia in 86 Chinese patients [31]. In AZA treatment for neuroimmunological disease, *NUDT15* c.415T variant showed a higher frequency of leukopenia (OR = 6.5, *p*= 0.003) and severe alopecia (OR = 41.9, *p* = 0.001) during treatment [32]. AZA-induced leukopenia was observed within 8 weeks of treatment in all patients with *NUDT15* c.415T/T genotypes. This study population did not differ in AZA dose among those with *NUDT15* genotypes, but the white blood cell counts were severely decreased in patients with a homozygous c.415T variant.

In dermatologic disease, *NUDT15* c.415C>T (p.R139C) variant was associated with a severe decrease in neutrophil count (<1500/mm^3^) in 56 Chinese patients [33]. All three patients with *NUDT15* c.415T/T had a neutrophil count of <1500/mm^3^ within 8 weeks of treatment. Therefore, *NUDT15* non-functional variants were also a risk factor for intolerability to thiopurine treatment in patients with autoimmune and rheumatic diseases.

In addition, AZA is used as an immunosuppressive therapy after organ transplantation. For this indication, only one case report was conducted to examine a kidney transplantation patient with a homozygous *NUDT15* p.139C variant [34]. This patient developed fever, leukopenia, and alopecia within 4 days of AZA initiation and was treated with mycophenolate mofetil.

## 3. Dose Adjustment Based on *NUDT15* Alleles

The CPIC guideline recommends thiopurine dosing based on the *NUDT15* diplotypes (Table 2). For malignant patients with *NUDT15* poor metabolizer diplotypes (*2/*2, *2/*3, and *3/*3), thiopurine drugs start in drastically reduced daily dose, i.e., 10 mg/m^2^ of 6-MP, to minimize toxicities. For patients with non-malignant conditions, the guideline recommends the use of an alternative non-thiopurine immunosuppressant therapy for *NUDT15* poor metabolizers.

Chang et al. reported the efficacy of adjusting the AZA dose based on the results of preemptive genotyping for common variants of *NUDT15* (rs116855232; p.R139C), *FTO* (rs79206939), and *TPMT* (rs1800460, rs1800462 and rs1142345; p.A154T, p.A80P, p.Y240C or Y240S, respectively) compared with non-genotyping group in 164 Korean IBD patients [35]. The heterozygotes of the genotyped patients received AZA 50 mg or 6-MP 25 mg daily, while the homozygotes were recommended to take alternative drugs. The genotyping group showed a lower frequency of myelosuppression (WBC count of <3000/mm^3^, platelet count of <1 × 10^6^/mm^3^, and hemoglobin level of <10 g/dL, which decreased to >2 g/dL) than the non-genotyping group (17% vs. 36%, *p* = 0.005). Furthermore, fewer thiopurine treatment discontinuations or dose reductions were required in the genotyping group. However, to the best of our knowledge, the efficacy of 6-MP initial dose adjustment based on the *NUDT15* variants to achieve favorable therapeutic outcomes has not been reported in any disease.

## 4. *NUDT15* Genotype Information in Drug Labels

Based on the above sections, data on the *NUDT15* genotype are useful for ensuring the safety of patients administered thiopurines, in addition to *TPMT* genotypes. In this context, the drug labels of AZA and 6-MP in some countries include *NUDT15* genotype information (Table 3). The labels of US FDA describe the consideration of *NUDT15* genotyping in patients experiencing severe myelosuppression. If patients with homozygous deficient alleles of either *TPMT* or *NUDT15*, alternative therapies (AZA) or dosage reduction (6-MP) should be considered. For patients with heterozygous deficient alleles of either *TPMT* or *NUDT15*, the drug labels indicate the recommendation for dosage reduction (AZA) or reduced dosage based on tolerability (6-MP). UK MHRA drug labels indicate an increased risk of toxicity among patients with inherited mutated *NUDT15* gene, and reduced dosage is generally required for these patients. Genotypic testing of *NUDT15* variants may be considered before initiating AZA and 6-MP therapies. A similar description was found in the Canadian and Australian drug labels of AZA. In addition, the Australian drug labels stipulate that Asians have a particular risk of *NUDT15* deficiency. Japanese drug labels specify the frequent occurrence of leukopenia among patients with the *NUDT15* p.R139C genotype, referring to published papers. In the AZA label, caution should be paid, such as considering the use of other drugs in patients with the *NUDT15* p.R139C genotype. Interestingly, the Singapore and Malaysia drug labels did not specify any warnings for patients with *NUDT15* genotypes. Because Asians have higher frequencies of the *NUDT15* p.R139C allele than European and African populations, the drug labels may be revised in the future.

## 5. Conclusions

*NUDT15* is a major predictive genetic marker of thiopurine tolerance in Asians. *NUDT15* c.415C>T (p.R139C) is a common variant and induces severe myelosuppression and hair loss; thus, patients with this variant require a dose reduction. However, the clinical significance of other low-frequency variants has not yet been fully evaluated. The functional effects are likely to vary depending on the *NUDT15* alleles, and determining the *NUDT15* diplotype could help in the development of a thiopurine therapy that is tailored to the needs of each patient. Further studies are needed to evaluate the clinical usefulness of determining the initial thiopurine dose based on preemptive *NUDT15* genotyping over the next decade.

## Figures and Tables

**Table 1:** Clinical impact of *NUDT15* alleles in Asians.

Allele	Variation	Amino Acid	Estimated Enzyme Activity	Disease	Clinical Impact	Reference
*2	55_56insGAGTCG 415C>T	V18_V19insGV R139C	Low	IBD ALL	AZA dose reduction Leukopenia and hair loss Leukopenia, 6-MP dose reduction	[12] [13] [14,15]
*3	415C>T	R139C	Low	IBD ALL	AZA dose reduction Leukopenia and hair loss Leukopenia, dose reduction	[12] [13] [10,14,16]
*4	416G>A	R139H	Intermediate	IBD	Leukopenia and hair loss (homo)	[13,17]
*5	52G>A	V18I	Intermediate	IBD ALL	Leukopenia 6-MP dose reduction (homo)	[13] [14]
*6	55_56insGAGTCG	V18_V19insGV	Intermediate			
*7	101G>C	R34T	Low	ALL	Dose reduction	[11]
*8	103A>G	K35E	Intermediate	ALL	Dose reduction (patient with *2/*8)	[11]
*9	50delGAGTCG	del17_18GV	Low			

IBD, inflammatory bowel disease; ALL, acute lymphoblastic leukemia; AZA, azathioprine; 6-MP, 6-mercaptopurine.

**Table 2:** Recommended initial treatment and dose adjustment of thiopurine therapy for *NUDT15* genotype based on the CPIC guidelines.

*NUDT15* Phenotype	Diplotypes	Mercaptopurine	Azathioprine
Intermediate	*1/*2 and *1/*3	Start with reduced starting doses (30–80% of normal dose) if normal starting doses is ≥75 mg/m^2^/day or ≥1.5 mg/kg/day and adjust the dose of 6-MP based on the degree of myelosuppression and disease-specific guidelines.	Start with reduced starting doses (30–80% of normal dose) if normal starting dose is 2–3 mg/kg/day and adjust the doses of AZA based on the degree of myelosuppression and disease-specific guidelines.
Possible intermediate metabolizer	*2/*4–*9 and *3/*4–*9		
Poor metabolizer	*2/*2, *2/*3, and *3/*3	Malignancy An Initial dose of 10 mg/m^2^/day should be used, and the dose should be adjusted based on the degree of myelosuppression and disease-specific guidelines. Non-malignancy Consider alternative nonthiopurine immunosuppressant therapy.	Malignancy Start with drastically reduced normal daily doses of AZA (reduce daily dose 10-fold) and then adjust the doses based on degree of myelosuppression and disease-specific guidelines. Non-malignancy Consider alternative nonthiopurine immunosuppressant therapy.

**Table 3:** Description of *NUDT15* polymorphisms in the drug labels of countries.

Country	Description of *TPMT* Polymorphisms	Description of *NUDT15* Polymorphisms in the Drug Labels	
		Azathioprine (AZA) *	6-Mercaptopurine (6-MP)
US	AZA: ○ 6-MP: ○	**Dosage and Administrations:** Patients with *TPMT* and/or *NUDT15* Deficiency: Consider testing for *TPMT* and *NUDT15* deficiency in patients who experience severe bone marrow toxicities. Homozygous deficiency in either *TPMT* or *NUDT15*: Because of the risk of increased toxicity, consider alternative therapies for patients who are known to have *TPMT* or *NUDT15* deficiency. Heterozygous deficiency in *TPMT* and/or *NUDT15*: Because of the risk of increased toxicity, dosage reduction is recommended in patients known to have heterozygous deficiency of *TPMT* or *NUDT15*. Patients who are heterozygous for both *TPMT* and *NUDT15* deficiency may require more substantial dosage reductions. (Also described in the Warnings section and Precaution section)	**Dosage and Administrations:** Dosage Modifications in Patients with *TPMT* and *NUDT15* Deficiency: Consider testing for *TPMT* and *NUDT15* deficiency in patients who experience severe myelosuppression or repeated episodes of myelosuppression. Homozygous Deficiency in either *TPMT* or *NUDT15*: Patients with homozygous deficiency of either enzyme typically require 10% or less of the recommended dosage. Reduce the recommended starting dosage in patients who are known to have homozygous *TPMT* or *NUDT15* deficiency. Heterozygous Deficiency in *TPMT* and/or *NUDT15*: Reduce the dose based on tolerability. Most patients with heterozygous *TPMT* or *NUDT15* deficiency tolerate the recommended dosage, but some require a dose reduction based on adverse reactions. Patients who are heterozygous for both *TPMT* and *NUDT15* may require more substantial dose reductions. (Also described in the Warnings and Precautions section)
UK	AZA: ○ 6-MP: ○	**Posology and method of administration:** Patients with *NUDT15* variant: Patients with inherited mutated *NUDT15* gene are at increased risk for severe AZA toxicity. These patients generally require dose reduction particularly those with homozygous *NUDT15* variants. Genotypic testing of *NUDT15* variants may be considered before initiating AZA therapy. In any case, close monitoring of blood counts is necessary. (Also described in the Special Warning and Precautions section)	**Posology and method of administration:** Patients with *NUDT15* variant: Patients with inherited mutated *NUDT15* gene are at increased risk for severe 6-MP toxicity. These patients generally require dose reduction; particularly those with homozygous *NUDT15* variants. Genotypic testing of *NUDT15* variants may be considered before initiating 6-MP therapy. In any case, close monitoring of blood counts is necessary. (Also described in the Special Warning and Precautions section)
Canada	AZA: ○ 6-MP: ○	**Dosage and Administrations:** Patients with *NUDT15* variant Patients with inherited mutated *NUDT15* gene are at increased risk for severe 6-MP toxicity. These patients generally require dose reduction, particularly those with homozygous *NUDT15* variants. Genotypic testing of *NUDT15* variants may be considered before initiating 6-MP therapy. In any case, close monitoring of blood counts is necessary. (Also described in the Warning and Precautions section and Pharmacokinetics section)	Not mentioned.
Australia	AZA: ○ 6-MP: ○	**Special Warnings and Precautions for Use:** *NUDT15* Testing Patients with inherited mutated *NUDT15* gene are at increased risk for severe thiopurine toxicity, such as early leukopenia and alopecia, from conventional doses of thiopurine therapy and generally require substantial dose reduction. Patients of Asian ethnicity are particularly at risk, due to the increased frequency of the mutation in this population. The optimal starting dose for heterozygous or homozygous deficient patients has not been established. Close monitoring of blood cell count is necessary. Genotypic and phenotypic testing of *NUDT15* variants should be considered before initiating thiopurine therapy in all patients (including pediatric patients) to reduce the risk of thiopurine-related severe leukocytopenia and alopecia, especially in Asian populations.	Not mentioned.
Japan	AZA: ○ 6-MP: X	**Important precautions:** It has been reported that patients with *NUDT15* Arg139Cys genetic polymorphisms are more likely to develop leukopenia after drug administration. Therefore, caution should be exercised, such as considering the use of other drugs. (Also described in the Other Precautions section)	**Other Precautions:** It has been reported that patients with *NUDT15* Arg139Cys genetic polymorphisms are more likely to develop leukopenia after drug administration.
Singapore	AZA: ○ 6-MP: ○	Not mentioned	Not mentioned
Malaysia	AZA: ○	Not mentioned	Label was not available.

* IMURAN labels. AZP, azathioprine; 6-MP, 6-mercaptopurine.

## Data Availability

Not applicable.
